# Supplementary material for: Epistasis at the cell surface: what is the role of Erg3 loss-of-function in acquired echinocandin resistance?
Source: mBio. 2025 Sep 9;16(10):e01419-25. doi: 10.1128/mbio.01419-25 (PMC12505910; doi:10.1128/mbio.01419-25)
Supplement: Supplemental material — Table S1. [file mbio.01419-25-s0001.docx]

**Supplementary**

**Table S1.** Unique variants identified between isolate A and isolate B in whole-genome sequencing analysis. For each variant, the amino acid change (a.a. change), nucleotide change (nt. change), consequence (type of mutation) , gene ID in the reference genome used in this study, and corresponding *S. cerevisiae* ortholog name (Sc. orth.) is given. The mutation in *ERG3* is highlighted in grey.

| **strain** | **a.a. change** | **nt. change** | **consequence** | **gene ID** | **Sc. orth.** |
| --- | --- | --- | --- | --- | --- |
| isolate A | N/D | Aat/Gat | missense_variant | CAGL0A00451g | *PDR1* |
| isolate A | T/S | Act/Tct | missense_variant | CAGL0C00209g |  |
| isolate A | S/F | tCt/tTt | missense_variant | CAGL0C00209g |  |
| isolate A | RSISSSISSSISSS/- | AGATCCATTTCTAGCTCCATTTCTAGCTCCATTTCTAGCTCC/- | inframe_deletion | CAGL0C00968g |  |
| isolate A | A/G | gCt/gGt | missense_variant | CAGL0C01078g |  |
| isolate A | -/HRX | -/CACAGACA | inframe_insertion,frameshift_variant | CAGL0C05467g | *MFG1* |
| isolate A | K/N | aaA/aaC | missense_variant | CAGL0D00462g | *ASH1* |
| isolate A | V/I | Gtt/Att | missense_variant | CAGL0D06732g |  |
| isolate A | P/X | ccT/cc | frameshift_variant | CAGL0D06732g |  |
| isolate A | Q/H | caA/caC | missense_variant | CAGL0D06732g |  |
| isolate A | SAAPSS/- | TCTGCCGCTCCATCTTCT/- | inframe_deletion | CAGL0F01463g | *TIR2* |
| isolate A | L/R | cTt/cGt | missense_variant | CAGL0G00105g |  |
| isolate A | K/N | aaG/aaC | missense_variant | CAGL0G00110g |  |
| isolate A | L/R | cTt/cGt | missense_variant | CAGL0G00110g |  |
| isolate A | Q/E | Caa/Gaa | missense_variant | CAGL0G10219g |  |
| isolate A | V/L | Gta/Cta | missense_variant | CAGL0G10219g |  |
| isolate A | -/EKD | -/GAGAAAGAC | inframe_insertion | CAGL0H02189g | *TMA23* |
| isolate A | S/SPAVAA | tca/tCACCCGCTGTGGCAGca | inframe_insertion | CAGL0I04466g | *BUD14* |
| isolate A | T/A | Act/Gct | missense_variant | CAGL0J01774g |  |
| isolate A | Q/QQQ | caa/caGCAACAa | inframe_insertion | CAGL0J06072g | *CBK1* |
| isolate A | GGL/G | ggTGGGCTt/ggt | inframe_deletion | CAGL0J09988g | *MTQ1* |
| isolate A | T/S | aCt/aGt | missense_variant | CAGL0J11968g |  |
| isolate A | Q/S | CAa/TCa | stop_gained | CAGL0J11968g |  |
| isolate A | M/I | atG/atA | missense_variant | CAGL0M00132g |  |
| isolate B | R/G | Aga/Gga | missense_variant | CAGL0C01056g |  |
| isolate B | G/D | gGt/gAt | missense_variant | CAGL0C03267g | *FPS1* |
| isolate B | M/X | aTg/ag | frameshift_variant | CAGL0D06732g |  |
| isolate B | L/* | tTg/tAg | stop_gained | CAGL0F01793g | *ERG3* |
| isolate B | -/GGGS | -/GGTGGCGGCTCC | inframe_insertion | CAGL0G05984g | *SPS100* |
| isolate B | D/N | Gat/Aat | missense_variant | CAGL0H01419g | *BFR2* |
| isolate B | M/T | aTg/aCg | missense_variant | CAGL0H10626g |  |
| isolate B | N/K | aaC/aaA | missense_variant | CAGL0I10362g |  |
| isolate B | I/T | aTa/aCa | missense_variant | CAGL0I10362g |  |
| isolate B | S/C | tCt/tGt | missense_variant | CAGL0J01774g |  |
| isolate B | SNANS/S | tcAAATGCAAACTCt/tct | inframe_deletion | CAGL0J01800g |  |
| isolate B | A/V | gCa/gTa | missense_variant | CAGL0J08569g | *OST3* |
| isolate B | V/A | gTc/gCc | missense_variant | CAGL0L00157g |  |
| isolate B | F/V | Ttc/Gtc | missense_variant | CAGL0L00157g |  |
| isolate B | V/A | gTa/gCa | missense_variant | CAGL0L00157g |  |
| isolate B | Y/S | tAc/tCc | missense_variant | CAGL0L11814g | *DNF1* |
| isolate B | M/I | atG/atA | missense_variant | CAGL0M00132g |  |
| isolate B | T/A | Aca/Gca | missense_variant | CAGL0M10527g |  |
